# Depth-Related Patterns and Physicochemical Drivers of Soil Microbial Communities in the Alpine Desert of Ngari, Xizang

**DOI:** 10.3390/microorganisms13122775

**Published:** 2025-12-05

**Authors:** Lan Wang, Ciren Quzong, Sang-Gyal Skal, Chengwei Mu, Yaqin Zhao, Bo Fang, Yuan Zhang, Zhiyong Yang, Erping Hei, Xin Yuan, Tsechoe Dorji

**Affiliations:** 1 Key Laboratory of Biodiversity and Environment on the Qinghai–Xizang Plateau, Ministry of Education, Xizang University, Lhasa 850000, China; wl_xzdx@163.com (L.W.); cirenquzong@itpcas.ac.cn (C.Q.); skalsanggyal@outlook.com (S.-G.S.); fangb1994@gmail.com (B.F.); yuansoua@163.com (Y.Z.); heichu20@163.com (E.H.); 2 School of Ecology and Environment, Xizang University, Lhasa 850000, China; 3 State Key Laboratory of Tibetan Plateau Earth System, Resources and Environment (TPESRE), Institute of Tibetan Plateau Research, Chinese Academy of Sciences, Beijing 100101, China; yangzy@itpcas.ac.cn (Z.Y.); yuanxin0503@163.com (X.Y.); 4 Naqu Alpine Grassland Ecosystem Field National Scientific Observation and Research Station, Naqu 852000, China; 5 Forestry and Grassland College, Xizang Agricultural and Animal Husbandry University, Nyingchi 860000, China; mcw0224@163.com (C.M.); zhaoyaqin0914@163.com (Y.Z.)

**Keywords:** soil microbial community, soil physicochemical drivers, functional potential, Qinghai–Xizang Plateau, alpine desert restoration

## Abstract

The Beishan region near Shiquanhe Town in Ngari, western Xizang (Tibet), represents a typical alpine desert ecosystem on the Qinghai–Xizang Plateau. However, depth-related patterns of soil microbial communities and their physicochemical controls remain insufficiently understood. Here, microbial community composition and functional attributes were examined across three soil horizons—topsoil (0–20 cm), subsoil (20–40 cm), and deep subsoil (40–60 cm)—sampled in May 2024 prior to artificial greening. High-throughput 16S rRNA and ITS sequencing combined with physicochemical analyses revealed clear vertical stratification: bacteria were dominated by Proteobacteria and Actinobacteriota, and fungi by Ascomycota. Bacterial diversity was higher in the topsoil, whereas fungal diversity exhibited a gradual increase with soil depth; however, these trends did not reach statistical significance (*p* > 0.05). Functional predictions indicated predominantly aerobic heterotrophic bacteria and a shift from pathogenic to saprotrophic fungi with depth. Multivariate analyses (RDA, CCA, BRT) consistently identified soil pH and moisture as fundamental habitat constraints, and organic carbon, available phosphorus, and available potassium as physicochemical drivers with nonlinear threshold responses. These results highlight soil pH, moisture, and nutrient status (N, P, K) as primary determinants of microbial community assembly and provide guidance for microbially informed ecological restoration in alpine desert ecosystems.

## 1. Introduction

In recent decades, arid regions across the globe have continued to expand, and projections suggest that by 2100, more than half of Earth’s land surface will be classified as drylands [1]. Consequently, soil degradation and desertification have emerged as major challenges in these ecosystems, manifested through the overexploitation of soil resources, declining soil quality, and the increasing frequency of dust storms [2,3]. These processes ultimately reduce soil fertility, accelerate vegetation loss, and substantially impair overall ecosystem functioning [4,5]. Among the various types of drylands, desert ecosystems are particularly fragile, with their stability and ecological functioning largely dependent on soil microorganisms—the essential drivers of biogeochemical cycling [6]. By decomposing litter and utilizing root exudates, soil microorganisms drive the cycling of key nutrients such as carbon, nitrogen, and phosphorus, thus sustaining plant nutrition and promoting ecosystem recovery and resilience [7,8,9]. In these fragile habitats, specific groups of soil microorganisms—such as nitrogen-fixing bacteria, phosphate-solubilizing bacteria, and plant growth-promoting fungi—play vital roles in improving nutrient availability, facilitating plant establishment, and enhancing their resilience to environmental stressors. For instance, two bacterial strains, SDQ-1 and MC-20, obtained from the Baiqi Desert steppe in Ningxia, were found to markedly promote both shoot and root growth of alfalfa (*Medicago sativa*) [10]. Similarly, plant growth-promoting bacteria isolated from the Namib Desert maintained their ability to secrete siderophores and to solubilize phosphate and potassium even under severe salinity and pH stress, underscoring their value in sustaining plant growth under extreme conditions [11]. The structure and functioning of soil microbial communities, in turn, provide valuable indicators of desert ecosystem health, as their diversity and stability strongly affect nutrient cycling and vegetation recovery [12]. Therefore, understanding the structure of desert soil microbial communities and the environmental factors shaping them is fundamental to evaluating the ecological impacts of desertification and to devising sustainable strategies for restoring and maintaining ecosystem functions.

Considerable progress has been achieved in China toward characterizing the microbial communities inhabiting desert soils. For instance, in the Qaidam Basin, the bacterial community is dominated by Proteobacteria, Bacteroidota, Actinobacteriota, and Firmicutes, with *Halomonas* showing the highest relative abundance. The diversity of microbial communities in this region is largely controlled by electrical conductivity and the content of water-soluble salts [13]. In the Hexi Corridor, bacterial communities are dominated by the phyla Proteobacteria, Actinobacteriota, Bacteroidota, and Firmicutes, whereas fungal assemblages are mainly composed of Ascomycota and Basidiomycota. Community diversity varies markedly across regions and is primarily driven by the combined effects of climate and soil physicochemical properties [14]. Similarly, in the Junggar Basin of Xinjiang, soil bacterial communities vary markedly across desert vegetation types, with Actinobacteriota, Proteobacteria, Acidobacteriota, and Bacteroidota being the dominant phyla; *Actinobacteriota* alone accounts for more than 50% of the total abundance. Community structure and diversity are largely governed by mean annual precipitation and soil organic carbon. [15]. Nevertheless, most existing studies have mainly concentrated on the taxonomic description communities, investigations into functional potentials, vertical (depth-related) variations, and quantitative effects of multiple physicochemical drivers remain limited. Such research gaps are especially evident in extreme environments, notably on the Qinghai–Xizang Plateau.

The Qinghai–Xizang Plateau is recognized as one of the world’s most representative cold and arid regions and is highly sensitive to both climatic variability and human activities. Previous studies have demonstrated that soil microbial communities on the Plateau are mainly shaped by soil pH, nitrogen levels, the carbon-to-nitrogen ratio, and soil texture [16,17]. However, little information is available regarding the microbial composition of its desert soils. Located in the Plateau’s western sector, the Ngari region features a harshly cold and arid climate, nutrient-deficient soils, persistent strong winds, and minimal rainfall [18,19], forming an ecosystem of remarkable fragility. Due to the loose soil structure and low organic matter content, vegetation recovery and ecological restoration in this region have proceeded slowly. In recent years, extensive greening and ecological restoration initiatives have prioritized soil improvement and vegetation rehabilitation as key components of environmental management programs in the Ngari region [20,21]. However, the structural characteristics and soil physicochemical drivers governing desert soil microbial communities in this region remain poorly understood, thereby limiting the scientific development of ecological restoration strategies. Accordingly, this study aimed to elucidate the composition and key physicochemical determinants of soil microbial communities across multiple soil depths in the Beishan region of Ngari, western Xizang, by integrating high-throughput sequencing with analyses of soil physicochemical properties. Specifically, this study addressed three questions: (1) How do microbial community structure and diversity vary along soil depth gradients? (2) Which physicochemical properties exert dominant influences on community composition, and what are their relative contributions? (3) How do microbial functional potentials respond to soil depth and the associated physicochemical changes? The findings are expected to provide a scientific basis for desertification control and ecological restoration in Ngari and to offer theoretical insights into the sustainable management of alpine arid ecosystems.

## 2. Materials and Methods

### 2.1. Study Area and Soil Sampling

**Figure 1:**
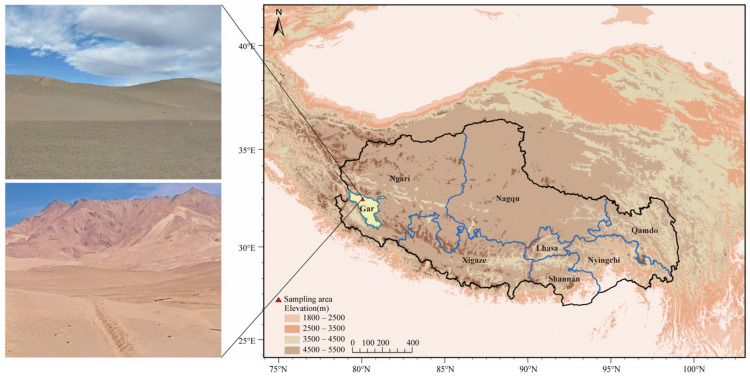
Geographic location of the study area in Beishan, Ngari Prefecture, western Xizang, on the western Qinghai–Xizang Plateau (**right**), and representative field photographs showing the alpine desert landscape of the sampling site (**left**).

The study area, located in Beishan near Shiquanhe Town, Ngari Prefecture, western Xizang (32.50° N, 80.05° E), lies on the western Qinghai–Xizang Plateau and represents a typical alpine desert ecosystem characterized by an extremely cold and arid climate (Figure 1). Recently, this region has been designated as a key area for large-scale greening and ecological restoration projects, making it an ideal site for studying soil microbial ecology within artificial restoration zones.

Sampling was conducted in May 2024, before the implementation of planned artificial greening activities, at a 2.67 ha experimental site with representative and homogeneous soil and vegetation conditions. According to topography, vegetation, and soil distribution, eight fixed quadrats (S1–S8) were established. Within each quadrat, soil samples were collected independently from three depth intervals—topsoil (0–20 cm, D1), subsoil (20–40 cm, D2), and deep subsoil (40–60 cm, D3)—using a five-point composite method. For each depth, soil was taken from the central portion of the designated interval while maintaining a 2–3 cm buffer from the upper and lower boundaries, and sterilized tools were used to avoid mixing between layers.

Each composite soil sample was divided into two subsamples: one for physicochemical analysis and the other for microbial community sequencing. Samples were preserved under refrigerated (4 °C) or frozen (–20 °C) conditions, depending on analytical requirements. The physicochemical analyses included measurements of soil pH, moisture content (MC), total nitrogen (TN), total phosphorus (TP), total potassium (TK), organic carbon (OC), organic matter (OM), alkali-hydrolyzable nitrogen (AN), available phosphorus (AP), and available potassium (AK). Detailed analytical procedures and corresponding references are summarized in Table A1.

### 2.2. DNA Extraction and PCR Amplification

Under sterile laboratory conditions, approximately 0.5 g of fresh soil from each sample was used for total DNA extraction with the DNeasy PowerSoil Kit (Qiagen, Hilden, Germany). The extracted DNA was checked for integrity on 1% agarose gels, and its concentration and purity were evaluated with a NanoDrop 2000 spectrophotometer (Thermo Fisher Scientific, MA, USA). To analyze bacterial communities, the V3–V4 region of the 16S rRNA gene was amplified with primers 338F and 806R, while fungal communities were examined by amplifying the ITS1 region using primers ITS1F and ITS2R. Each PCR reaction (25 μL) contained 2× Taq PCR Master Mix (Vazyme, Nanjing, China), 0.2 μmol·L^−1^ of each primer, around 10 ng of template DNA, and nuclease-free water. The thermal cycling program began with denaturation at 95 °C for 3 min, followed by 35 cycles of 95 °C for 30 s, 55 °C for 30 s, and 72 °C for 45 s, and ended with a final extension at 72 °C for 10 min. The resulting PCR products were purified using magnetic beads and sequenced on an Illumina MiSeq platform (Illumina, San Diego, CA, USA) with paired-end reads.

### 2.3. Sequence Processing and Bioinformatics Analysis

Raw sequencing reads were first quality-checked using FastQC (v0.11.9) and subsequently trimmed with Trimmomatic (v0.39) to remove low-quality bases and adapter sequences. High-quality reads were processed through the DADA2 pipeline (v1.22.0, R package) for denoising, merging, and chimera removal, resulting in an amplicon sequence variant (ASV) table. For bacterial datasets, chloroplast and mitochondrial sequences were filtered out to retain only authentic bacterial reads. Taxonomic assignment was carried out using the SILVA v138 database for bacterial 16S rRNA genes and the UNITE v8.3 database for fungal ITS sequences. Functional annotation of bacterial communities was performed using FAPROTAX v1.2.4, while fungal functional groups were classified according to the FUNGuild database (v2021.1). Only annotations with confidence levels of “probable” or “highly probable” were retained for downstream analyses.

### 2.4. Statistical Analyses

All statistical analyses were conducted in R version 4.4.3 using packages including vegan, phyloseq, and gbm. Soil physicochemical parameters and microbial α-diversity indices were first tested for normality and homogeneity of variance. Parametric data were analyzed using one-way ANOVA followed by Tukey’s HSD post hoc test (with *p*-values adjusted using the false discovery rate, FDR), whereas nonparametric data were evaluated with the Kruskal–Wallis test. β-diversity was calculated based on Bray–Curtis distances and visualized through principal coordinate analysis (PCoA). Group differences were assessed by PERMANOVA (999 permutations) and betadisper tests. Correlations among microbial diversity, functional groups, and environmental factors were evaluated using Spearman’s rank correlation, and significance levels were adjusted using the Benjamini–Hochberg FDR correction. Correlation coefficients were summarized in a matrix, where significance was denoted as *p* < 0.05 (*) and *p* < 0.01 (**). Relationships between community composition and environmental variables were examined via redundancy analysis (RDA) or canonical correspondence analysis (CCA), with the appropriate model determined based on detrended correspondence analysis (DCA). The significance of environmental variables was tested using the envfit function (999 permutations), and variables with variance inflation factors (VIF) > 10 were excluded. To identify major environmental drivers, boosted regression tree (BRT) models were constructed (n.trees = 1000, learning rate = 0.01, interaction depth = 2, bag fraction = 0.75). The relative influence (%) of each variable was quantified to indicate its importance, and partial dependence plots (PDPs) were generated to visualize nonlinear and threshold relationships between key environmental variables and microbial diversity or functional indices.

## 3. Results

### 3.1. Physicochemical Characteristics of Soils at Different Depths in Beishan, Ngari

The desert soils of Beishan in Ngari were characterized by strong alkalinity, nutrient scarcity, and low moisture availability (Table 1). Across the three sampled horizons, soil pH remained highly stable (*p* > 0.05), varying only within a narrow range of 9.19 to 9.29, indicating persistent alkalinity throughout the soil profile. Similarly, TN, TK, AN, and AP exhibited no statistically detectable differences among horizons (*p* > 0.05). In contrast, TP, OC, OM, and AK exhibited clear depth-related declines (*p* < 0.05), with the highest concentrations in the topsoil followed by progressive reductions toward the deep subsoil. For instance, OC dropped from 1.73 g·kg^−1^ in the topsoil to 1.32 g·kg^−1^ in deep subsoil, while AK decreased from 99.72 mg·kg^−1^ to 45.66 mg·kg^−1^. MC followed a similar trend (*p* < 0.05), remaining considerably higher in the topsoil than in deeper horizons. Overall, the upper horizons contained substantially greater moisture and nutrient reserves, consistent with higher organic matter inputs and greater root activity near the surface. In contrast, deeper horizons were markedly drier and more nutrient-poor, consistent with the pronounced desertification characteristic of alpine dryland soils on the Qinghai–Xizang Plateau.

### 3.2. Composition and Abundance of Microbial Communities at Different Soil Depths in Beishan, Ngari

**Figure 2:**
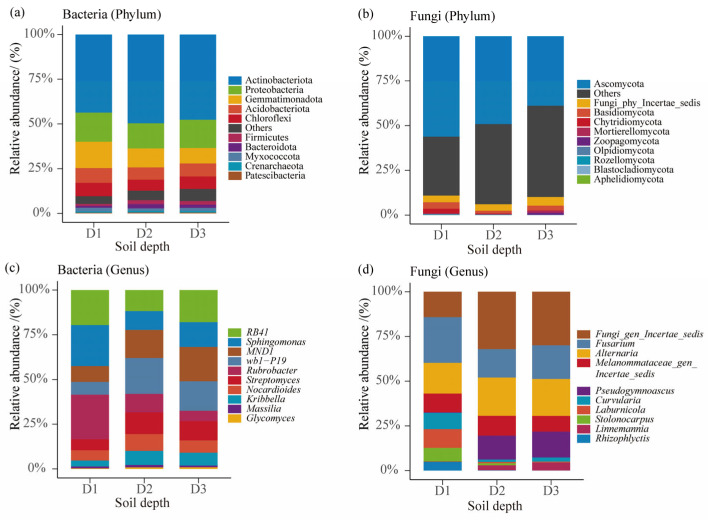
Relative abundance of soil bacterial and fungal communities at the phylum and genus levels across different soil depths. (**a**) Bacterial community composition at the phylum level. (**b**) Fungal community composition at the phylum level. (**c**) Bacterial community composition at the genus level. (**d**) Fungal community composition at the genus level.

Figure 2 presents the composition of bacterial and fungal communities at different soil depths in Beishan, Ngari, summarized at the phylum and genus levels. At the phylum level (Figure 2a), bacterial assemblages were dominated by Actinobacteriota and Proteobacteria, followed by Chloroflexi, Gemmatimonadota, and Bacteroidota—phyla commonly associated with arid soil environments. Although the overall bacterial composition remained largely consistent across the three depths, slight fluctuations in relative abundance were detected. Notably, Proteobacteria were slightly more abundant in the topsoil than in deeper horizons. For fungi (Figure 2b), Ascomycota overwhelmingly dominated across all depths, showing the highest relative abundance in the topsoil. Basidiomycota and Mortierellomycota were also important contributors, with Mortierellomycota exhibiting a gradual increase with depth—a pattern consistent with the prevalence of saprotrophic fungi in deeper, organic-poor soils. At the genus level (Figure 2c), major bacterial taxa included RB41, Sphingomonas, *norank_f__norank_o__norank_c__Subgroup_6*, Blastococcus, and Solirubrobacter, all of which were distributed throughout the soil profile. RB41 and Sphingomonas were more enriched in the topsoil, whereas Gaiella and Nocardioides increased with depth, suggesting a shift in dominant bacterial taxa along the vertical gradient. Regarding fungal taxa (Figure 2d), Aspergillus, Penicillium, Solicoccozyma, Alternaria, Fusarium, and Mortierella were the principal genera. Yeast-like taxa such as Solicoccozyma and Malassezia were more abundant in the topsoil and subsoil, whereas filamentous genera, including Fusarium and Mortierella, exhibited notable enrichment toward the deep subsoil. Overall, bacterial communities exhibited relatively stable compositions with only minor depth-related variation, whereas fungal assemblages showed more pronounced vertical differentiation. Taken together, these results indicate that soil depth exerts a stronger influence on fungal than on bacterial community structure in the Beishan alpine desert ecosystem.

**Figure 3:**
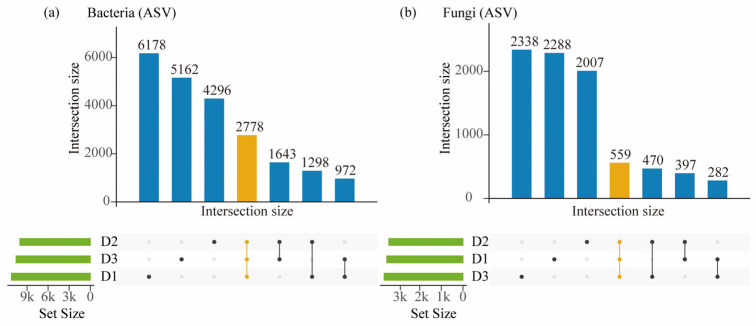
Set sizes and intersection patterns of bacterial (**a**) and fungal (**b**) ASVs across the topsoil (D1), subsoil (D2), and deep subsoil (D3). Filled dots and connecting lines denote the soil horizons contributing to each intersection, and the green bars represent the ASV set size of each individual horizon. The yellow bars and yellow dots highlight the intersection shared among all three horizons (D1, D2, and D3).

As illustrated in Figure 3, bacterial communities exhibited a high degree of overlap among soil depths. The number of ASVs was highest in the topsoil (D1) and subsoil (D2), with approximately 2700 ASVs shared among all three horizons (D1–D3), indicating strong compositional similarity and stability along the vertical soil profile. In contrast, fungal communities exhibited markedly fewer shared ASVs, with only about 700 ASVs detected across all depths, reflecting stronger depth-dependent differentiation and higher community specificity. Taken together, these results indicate that bacterial communities remain relatively stable throughout the soil profile, whereas fungal assemblages display higher spatial heterogeneity and more pronounced vertical stratification.

### 3.3. Microbial Community Diversity at Different Soil Depths in Beishan, Ngari

**Figure 4:**
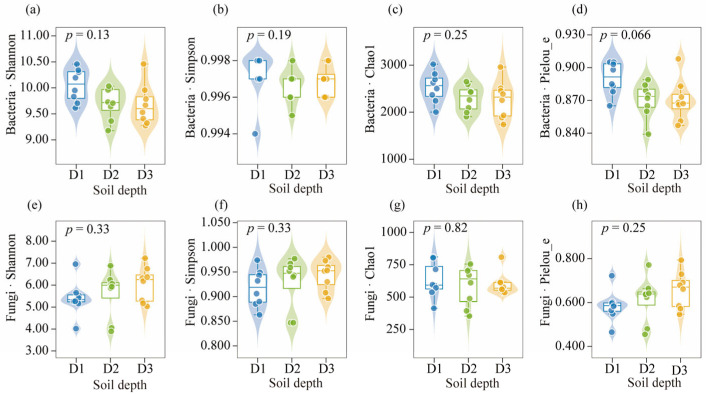
Comparison of α-diversity indices of soil bacterial and fungal communities across different soil depths. (**a**–**d**) Shannon, Simpson, Chao1, and Pielou_e indices of bacterial communities. (**e**–**h**) Shannon, Simpson, Chao1, and Pielou_e indices of fungal communities. *p*-values indicate the significance of differences among soil depths. The violin plots show the data distribution, the boxes represent the interquartile range with the median, and the dots indicate individual sample values.

Figure 4 illustrates the variation in α-diversity indices of bacterial and fungal communities across soil depths. For bacterial communities, the Shannon index was highest in the topsoil (10.05 ± 0.32) and declined slightly with depth. The Simpson index remained consistently high across all depths, with no statistically detectable difference (*p* > 0.05). Similarly, the Chao1 richness index decreased from 2533.17 ± 326.06 in the topsoil to 2264.64 ± 400.73 in the deep subsoil, while the Pielou evenness index declined slightly from 0.89 ± 0.01 to 0.87 ± 0.02. Although all bacterial diversity indices showed a downward trend with depth, no statistically detectable variation was observed. In contrast, fungal communities showed an opposite pattern. The Shannon index increased slightly with depth, from 5.41 ± 0.80 in topsoil to 6.04 ± 0.80 in the deep subsoil, while the Simpson index exhibited a similar but modest upward trend. The Chao1 index remained relatively constant across depths and was generally lower than that of bacteria. The Pielou evenness index also rose slightly, from 0.58 ± 0.07 to 0.66 ± 0.08, suggesting a weak increase in evenness with depth. However, similar to bacterial patterns, these depth-related differences did not reach statistical significance, as no statistically detectable variation was identified (*p* > 0.05). Overall, bacterial communities tended to exhibit higher diversity and richness in the topsoil, whereas fungal diversity increased slightly with depth. The absence of statistically detectable differences indicates that microbial α-diversity remained relatively stable across the vertical soil profile in the Beishan desert ecosystem.

**Figure 5:**
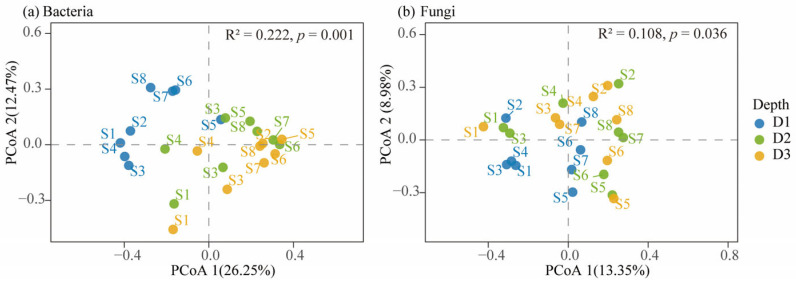
Principal coordinates analysis (PCoA) of soil bacterial (**a**) and fungal (**b**) communities across different soil horizons based on Bray–Curtis distances. (**a**) PCoA of bacterial communities. (**b**) PCoA of fungal communities. Each point represents a soil sample, and colors indicate soil horizons (D1: topsoil, 0–20 cm; D2: subsoil, 20–40 cm; D3: deep subsoil, 40–60 cm). R^2^ and *p*-values are derived from PERMANOVA (Adonis) tests.

Figure 5 presents PCoA results based on Bray–Curtis dissimilarities, illustrating depth-related variation in microbial community composition across topsoil, subsoil, and deep subsoil in Beishan, Ngari. For bacterial communities (Figure 5a), the first two principal coordinates (PCoA1 and PCoA2) explained 26.35% and 12.43% of the total variance, respectively. Samples from different depths formed distinct clusters in the ordination space, indicating a clear vertical differentiation in bacterial community structure. PERMANOVA confirmed that soil depth significantly influenced bacterial composition (R^2^ = 0.222, *p* = 0.001), whereas betadisper analysis detected no significant difference in within-group dispersion (*p* = 0.95), suggesting that the separation among depth horizons was primarily driven by compositional turnover rather than differences in dispersion. For fungal communities (Figure 5b), PCoA1 and PCoA2 accounted for 13.35% and 8.98% of the total variance, respectively. Although a depth-related separation was also observed, the clustering pattern was less distinct than in bacterial communities. PERMANOVA revealed a weaker but significant effect of soil depth (R^2^ = 0.108, *p* = 0.046), and betadisper again indicated no significant difference in dispersion (*p* = 0.183). Overall, both bacterial and fungal β-diversity displayed depth-associated patterns, with bacterial assemblages exhibiting stronger vertical stratification and greater sensitivity to depth-related environmental gradients. These findings indicate that bacterial communities in Beishan desert soils respond more strongly to depth-related changes than fungal communities, consistent with their differing ecological strategies under cold and arid conditions.

### 3.4. Functional Characteristics of Microbial Communities at Different Soil Depths in Beishan, Ngari

**Figure 6:**
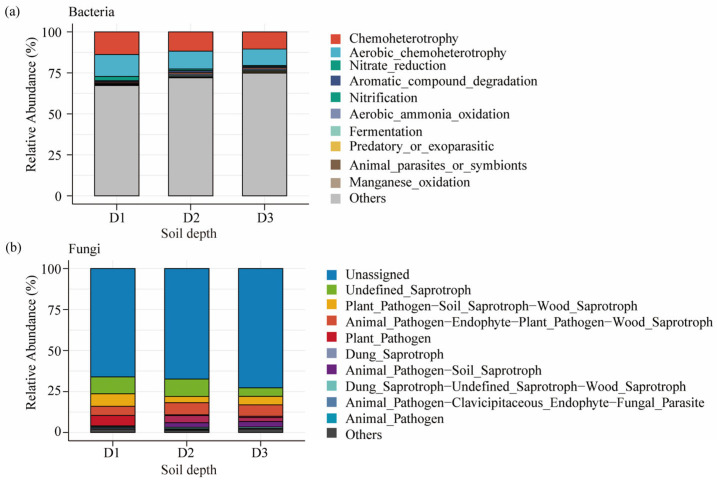
Predicted functional composition of soil bacterial (**a**) and fungal (**b**) communities across different depths. (**a**) Functional annotation of bacterial communities based on the FAPROTAX database. (**b**) Functional annotation of fungal communities based on the FUNGuild database.

Figure 6 illustrates the predicted functional profiles of bacterial (Figure 6a) and fungal (Figure 6b) communities across soil depths in Beishan, Ngari. In the bacterial community, chemoheterotrophy (CH) and aerobic chemoheterotrophy (ACH) were consistently dominant, together accounting for more than 80% of the total predicted functions at all depths (CH: 86.13%, 86.56%, and 84.48%; ACH: 85.69%, 85.65%, and 82.62% in the topsoil, subsoil, and deep subsoil, respectively). This pattern indicates that bacterial communities in this alpine desert are largely dependent on aerobic heterotrophic metabolism and maintain a functionally stable structure along the vertical soil profile. In contrast, minor functional groups such as fermentation and nitrate reduction exhibited moderate increases with depth. Notably, nitrate reduction increased from 3.43% in the topsoil to 4.97% in the deep subsoil, accompanied by enrichment in reductive metabolic pathways such as nitrate respiration, indicating an increased representation of anaerobic functions at greater depths.

For fungi, functional composition showed more pronounced depth-related shifts. The proportion of plant-pathogenic taxa was highest in the topsoil (77.61%) and decreased progressively with depth (66.77% and 59.99% in the subsoil and deep subsoil, respectively). Conversely, saprotrophic fungi—particularly dung saprotrophs—became increasingly dominant with depth, rising from 2.35% in the topsoil to 7.73% in the deep subsoil. The subsoil also contained a relatively higher proportion of unclassified or ecologically undefined taxa, suggesting the presence of groups whose ecological functions remain uncertain.

Collectively, these results reveal clear vertical stratification in microbial functional organization in Beishan desert soils: bacterial functions remained largely stable and predominantly aerobic, whereas fungal communities exhibited more pronounced depth-dependent functional shifts, consistent with their greater ecological plasticity under extreme alpine desert conditions.

### 3.5. Relationship Between Microbial Communities and Environmental Factors Across Soil Depths in Beishan, Ngari

**Figure 7:**
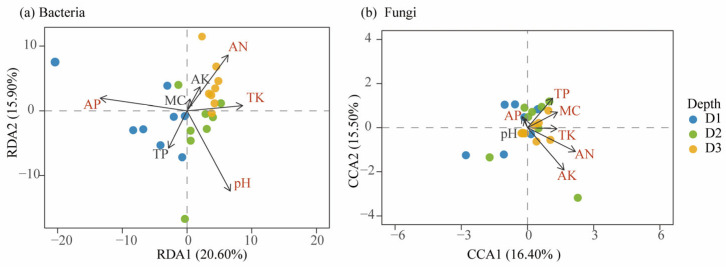
Ordination analysis of soil microbial communities and soil physicochemical factors at different soil depths. (**a**) Redundancy analysis (RDA) of bacterial community composition; (**b**) Canonical correspondence analysis (CCA) of fungal community composition. Samples are represented by points, with colors and shapes denoting distinct soil depths (D1: topsoil, 0–20 cm; D2: subsoil, 20–40 cm; D3: deep subsoil, 40–60 cm). Arrows denote significant soil physicochemical factors, including pH, TP, TK, MC, AK, AN, and AP. The length and orientation of the arrows represent the strength and direction of correlations between soil physicochemical factors and the ordination axes.

Figure 7 presents the results of RDA and CCA, illustrating the relationships between soil microbial community composition and soil physicochemical factors in Beishan, Ngari. For the bacterial communities (Figure 7a), the first two RDA axes explained 48.74% of the total variance in community structure. Although the overall model did not reach statistical significance (*p* = 0.096), the ordination still revealed clear ecological patterns. In comparison, the CCA model accounted for a smaller proportion of the variance (32.94%) and showed weaker overall performance (*p* = 0.162). Given its higher explanatory power, RDA was used as the primary ordination method to interpret bacterial–environment relationships. The RDA results indicated that soil pH, AN, and AP were significantly correlated with bacterial community composition (FDR-adjusted *p* < 0.05), with pH exhibiting the strongest explanatory power (r^2^ = 0.77, p.adj = 0.007). Although TK did not meet the significance threshold (p.adj = 0.056), it may represent a secondary environmental contributor to bacterial community variation.

For the fungal community (Figure 7b), DCA yielded a gradient length of 4.40 for the first axis—greater than 4.00—indicating a unimodal response to soil physicochemical factor gradients and thereby justifying the use of CCA. The CCA results showed that AN was the most influential factor shaping fungal community structure (r^2^ = 0.857, p.adj = 0.007), followed by TP, TK, AP, and AK, all significant after FDR correction (p.adj < 0.05). In contrast, pH and MC showed weaker associations with fungal community structure and did not exhibit statistically detectable correlations.

Overall, soil nutrient availability—particularly nitrogen and phosphorus—was identified as the primary driver of microbial community differentiation in Beishan desert soils. Bacterial and fungal assemblages responded differently to the same soil physicochemical factors, highlighting contrasting ecological strategies and functional redundancies and reflecting their distinct adaptive mechanisms under the nutrient-limited and environmentally variable conditions of this alpine desert ecosystem.

Table 2 summarizes the correlations among soil physicochemical factors, microbial diversity indices, and functional groups. In bacterial communities, the Simpson index was significantly positively correlated with TP (r = 0.42, *p* < 0.05), while the Pielou evenness index showed a similar positive correlation (r = 0.45, *p* < 0.05), indicating that phosphorus availability is closely associated with bacterial community stability and evenness. In contrast, fungal diversity exhibited stronger associations with soil moisture and nitrogen availability. The Simpson index of fungi was significantly positively correlated with MC (r = 0.35, *p* < 0.05) and TN (r = 0.40, *p* < 0.05), while the Chao1 richness index was also positively correlated with AK (r = 0.02, *p* < 0.05). Although the fungal Pielou evenness index also showed positive but statistically undetectable correlations with MC, TN, and OM, the overall pattern indicated that fungal community diversity responded more sensitively to variations in soil moisture and nutrient availability than bacterial diversity. Collectively, these results suggest that bacterial diversity in Beishan desert soils is more strongly associated with phosphorus availability, whereas fungal diversity is more closely linked to soil moisture and nitrogen status, reflecting differing ecological strategies and resource utilization patterns between the two microbial groups.

**Figure 8:**
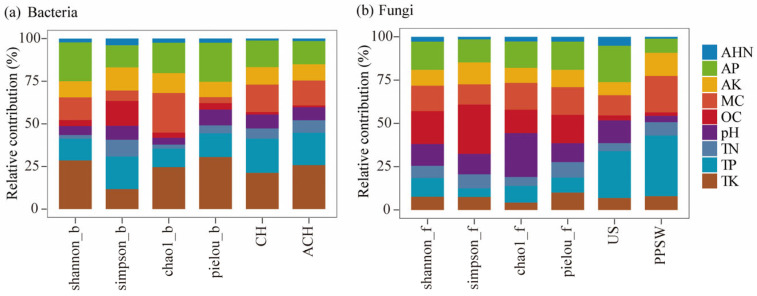
Explanatory contributions of different soil physicochemical factors to bacterial (**a**) and fungal (**b**) community α-diversity and functional traits. (**a**) Bacterial α-diversity indices (Shannon, Simpson, Chao1, and Pielou) and functional traits (chemoheterotrophy, CH; aerobic chemoheterotrophy, ACH). (**b**) Fungal α-diversity indices (Shannon, Simpson, Chao1, and Pielou) and functional traits (undefined saprotrophs, US; plant-pathogen–soil–wood saprotrophs, PPSW).

Figure 8 summarizes the results of BRT modeling, which was used to quantify the relative influence of soil variables on microbial diversity and functional composition in the Beishan region of Ngari Prefecture. For bacterial communities, TK, AP, and MC were identified as the principal controlling factors. Among them, TK contributed more than 25% to the total explanatory power and accounted for 30.58% of the variance in the Pielou evenness index, indicating that potassium availability, together with soil moisture and phosphorus supply, was closely associated with bacterial community stability. In contrast, fungal diversity was mainly regulated by soil pH and OC. pH exhibited the strongest explanatory power for the Chao1 richness index (25.35%), while OC significantly accounted for variations in Shannon, Simpson, and Pielou indices, suggesting that fungal assemblages were more responsive to changes in soil acidity–alkalinity and carbon availability. At the functional level, bacterial communities were dominated by chemoheterotrophic and aerobic chemoheterotrophic taxa, both of which were strongly associated with TK and TP. Fungal functional groups, in contrast, were primarily associated with TP, AP, and MC. Undefined saprotrophs were mainly linked to phosphorus availability (TP and AP), whereas more complex trophic guilds showed strong associations with TP and MC, highlighting the importance of nutrient availability in shaping fungal functional organization.

**Figure 9:**
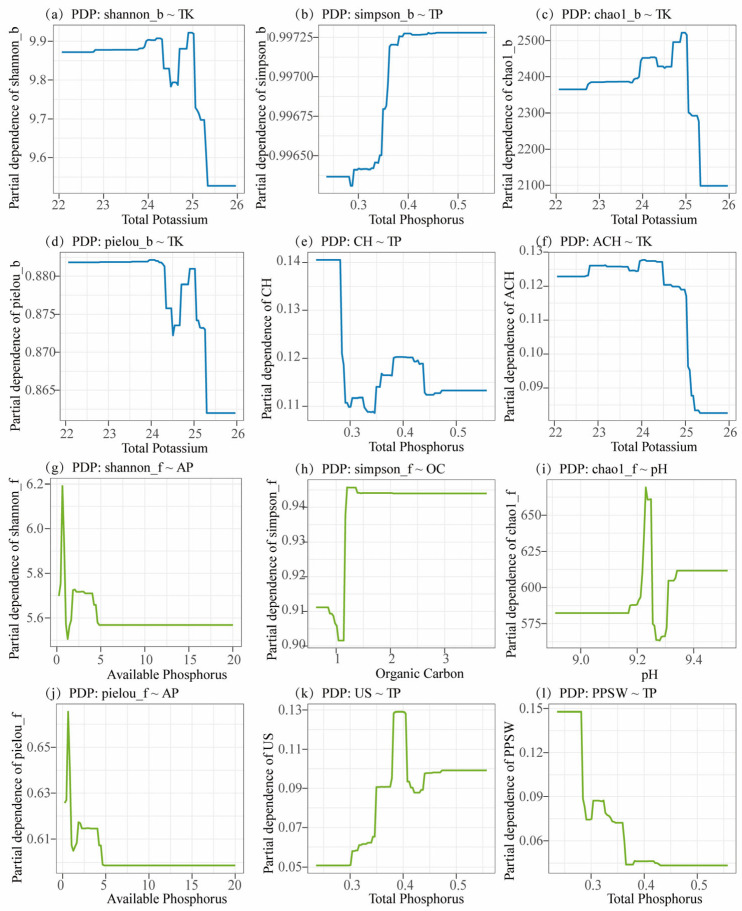
Partial dependence plots (PDPs) showing the marginal effects of key soil physicochemical factors on microbial diversity indices and functional traits. (**a**–**f**) Responses of bacterial α-diversity indices (Shannon, Simpson, Chao1, Pielou) and functional traits (chemoheterotrophy, CH; aerobic chemoheterotrophy, ACH) to TK and TP. (**g**–**l**) Responses of fungal α-diversity indices (Shannon, Simpson, Chao1, Pielou) and functional groups (undefined saprotrophs, US; plant-pathogen–soil–wood saprotrophs, PPSW) to OC, pH, AP, and total phosphorus TP.

PDP analysis further revealed nonlinear and threshold-type responses of microbial indices to these soil physicochemical factors (Figure 9). Bacterial Shannon, Chao1, and Pielou indices declined sharply once TK exceeded approximately 25 g·kg^−1^, implying that excessive potassium might have limited bacterial diversity. TP promoted increases in Simpson and CH indices at low to moderate levels (0.35–0.40 g·kg^−1^), beyond which the effects tended to plateau or decline. For fungi, the Simpson index exhibited a unimodal response to increasing OC, whereas the Chao1 index peaked at approximately pH 9.2. In contrast, the Shannon and Pielou indices increased sharply with AP at low concentrations before reaching saturation. Overall, the BRT and PDP analyses showed that potassium, phosphorus, moisture, pH, and carbon availability jointly contributed to variation in microbial diversity and functional structure in Beishan desert soils, exhibiting clear nonlinear and threshold-dependent response patterns. These results highlight the pronounced sensitivity of soil microbial communities to shifts in nutrient and moisture conditions under the extreme alpine desert environment of the Beishan region, Ngari Prefecture.

## 4. Discussion

### 4.1. Vertical Distribution Characteristics of Alpine Desert Soil Microbial Communities

No statistically detectable differences in α-diversity indices for either bacteria or fungi were observed across soil depths in the Beishan area, Ngari Prefecture, western Xizang (Tibet) (*p* > 0.05; Figure 4). These findings indicate that overall microbial diversity remained relatively stable along the desert soil profile under the strong abiotic constraints of this arid alpine environment. In contrast, both bacterial and fungal communities exhibited clear depth-related variation in community structure and metabolic potential. This vertical stratification suggests that even subtle shifts in soil physicochemical conditions with depth can influence microbial community assembly and functional capabilities. Depth-associated differences in resource inputs and microhabitat properties—such as greater organic inputs and disturbance near the surface versus more stable hydrothermal conditions at depth—may contribute to the observed vertical organization of microbial communities.

The bacterial flora was largely composed of Proteobacteria and Actinobacteriota, a dominance pattern consistent with previous findings from desert ecosystems worldwide [22,23,24,25]. Notably, bacterial α-diversity was highest in the topsoil (0–20 cm) and declined or remained relatively stable with increasing depth, a distribution pattern consistent with previous observations in other arid ecosystems [26,27,28]. This pattern is consistent with evidence from other arid ecosystems, where stronger rhizosphere effects, pronounced soil microstructural heterogeneity, and episodic nutrient inputs from precipitation and aeolian deposition collectively contribute to higher bacterial richness and evenness in the topsoil [29,30,31,32]. In addition, the relatively high availability of nutrients and micronutrients in surface soils, which was the case in our study (Table 1), might have reduced the strength of single-strategy environmental filtering and facilitated the coexistence of functionally complementary taxa, and might shape community strategy composition as the microbial life-history theories—such as the oligotrophic–copiotrophic continuum and the C–S–R framework—emphasizes [33]. In contrast, deeper soils lack direct root-derived inputs, are more limited in carbon and nutrients, and experience weaker hydrothermal fluctuations. As a result, they tend to be dominated by oligotrophic taxa adapted to resource-limited conditions, leading to reduced or more stable diversity with depth.

Fungal communities were largely dominated by Ascomycota, a phylum comprising many saprotrophic and yeast-like taxa. These fungi are well adapted to arid and stressful environments [34] and commonly associated with the decomposition of organic residues and the maintenance of nutrient cycling in desert soils elsewhere [35,36,37]. In contrast to bacteria, fungal α-diversity tended to increase with soil depth in our study, a pattern that was also observed in other arid and semi-arid ecosystems [38,39,40]. This trend is often attributed to more stable moisture and temperature conditions, as well as reduced root-derived inputs and pathogen pressure in deeper soils, which together lead to competitive release and favor fungal groups frequently found in arid or resource-limited environments—such as saprotrophs, dark septate endophytes, and various yeast-like taxa [41,42]. Fungal communities in the surface soil were enriched with plant-associated, pathogenic, and opportunistic taxa in our study. These groups were more exposed to disturbances and short-term resource pulses, which could disrupt community balance and lead to lower evenness [43,44]. Compared with high-litter forest soils or intensively managed agricultural soils [45,46,47,48,49], the Beishan region’s strong alkalinity (pH > 9), low organic carbon and available phosphorus, severe aridity, and persistent wind erosion impose strong environmental filtering. Under these constraints, saprotrophic and stress-tolerant fungal groups—often enriched in deeper soils—tend to be favored.

### 4.2. Driving Mechanisms of Physicochemical Factors on Alpine Desert Soil Microbial Communities

Analyses integrating RDA/CCA, BRT, and PDP approaches revealed that the microbial community structures were jointly regulated by soil pH, MC, OC, and phosphorus–potassium availability (AP/TP and AK/TK). The influence of these variables followed nonlinear trends and displayed clear threshold responses (Figure 8 and Figure 9; Table 2). Among these variables, pH emerged as one of the influential chemical variables that affect soil bacteria community in Beishan soils (BRT relative influence for bacteria >25%), as was widely recognized as a dominant driver of bacterial diversity and community composition in arid and desert ecosystems elsewhere [50,51]. Under strongly alkaline conditions, pH can exert marked filtering effects by reducing nutrient availability, disturbing cellular membrane homeostasis, and altering the NH_4_^+^/NH_3_ balance [52]. In contrast, fungi often show weaker apparent responses to pH, likely due to the protective properties of their cell walls and their capacity to regulate the surrounding microenvironment through the secretion of organic acids [53]. Moisture content represents the primary limiting factor for microbial communities in our study, consistent with previous findings that water availability governs microbial activity, substrate diffusion, and nutrient cycling in arid and semiarid ecosystems [54,55]. In fact, PDP curves in our study further revealed a clear moisture threshold: under low-moisture conditions, OC decomposition and AP release were constrained, whereas once moisture content exceeded a critical value, both diversity and functional metrics increased substantially. These results suggest that higher moisture strongly enhances the positive effects of nutrient availability on microbial communities. In addition, bacterial diversity indexes—particularly Pielou’s evenness—showed a “rapid increase at low AP levels followed by saturation” pattern, with an estimated threshold around AP ≈ 25 mg·kg^−1^, reflecting clear phosphorus limitation in the bacterial community [56]. In contrast, fungal functional groups exhibited more unimodal responses to total and available phosphorus, which were consistent with previous findings. For example, Kang et al. reported that increases in AP enhanced fungal diversity up to an intermediate level, beyond which the effect plateaued in desert grasslands [57].

In summary, the structure of soil microbial communities in the alpine desert region appeared to be jointly associated with fundamental edaphic conditions—particularly soil pH and moisture—and with nutrient-related factors such as OC, AP, and AK, which were also reflected in TP and TK contents. Although multicollinearity among variables was minimized through VIF screening, the relative influence values estimated from the BRT models should not be interpreted as direct evidence of causation. Therefore, these findings should be considered indicative rather than conclusive, and further validation through studies with larger and more stratified sampling designs—ideally incorporating additional parameters such as soil salinity, climatic variables, and controlled experimental approaches—would be beneficial.

### 4.3. Indicative Potential and Research Perspectives on Microbial Ecological Processes Under the Contexts of Alpine Desert Ecosystem Restoration

This study provides initial insights into how microbial communities vary under the combined constraints of strong alkalinity (pH > 9), low organic carbon, and limited available phosphorus and potassium in an alpine desert soil from the Beishan area, Ngari Prefecture, western Xizang. While depth-related patterns in microbial diversity and functional potential were evident, these findings are exploratory and should be interpreted cautiously given the single sampling event. The observed associations between soil physicochemical properties and microbial communities may offer preliminary directions for future research on the potential indicative value of microorganisms during ecological restoration, rather than providing firm evidence at this stage. These results, therefore, serve as a starting point for developing hypotheses to be tested in future experimental or long-term monitoring studies.

Building on these exploratory findings, several research avenues deserve further attention: (1) Long-term and multi-season monitoring to evaluate whether the vertical microbial patterns identified here are stable through time and across climatic variability. (2) Controlled experiments manipulating soil pH, moisture, and nutrient availability to clarify the causal relationships underlying microbial community responses. (3) Microbial indicator screening aimed at identifying taxa or functional attributes that may hold predictive or diagnostic value during ecological restoration processes. (4) Broader spatial comparisons across different cold-desert environments to determine the generality of the microbial–environment relationships observed in this study.

## 5. Conclusions

(1) Microbial communities in the alpine desert soils exhibited distinct vertical stratification, with higher bacterial diversity near the surface and increasing fungal diversity with depth.

(2) Soil pH, moisture content, and key nutrients (OC, AP, AK) were strongly associated with microbial community composition and structure, showing nonlinear and threshold-like relationships.

(3) Bacterial functions were relatively consistent across depths, while fungal functions shifted toward more saprotrophic and stress-tolerant groups in deeper layers. These findings provide baseline insights into microbial ecology in high-altitude arid soils.

## Figures and Tables

**Table 1:** Physicochemical properties of soils at different depths.

Depth	pH	MC	TN	TP	TK	OC	OM	AN	AP	AK
D1	9.19 ± 0.12 a	5.51 ± 0.7 a	0.2 ± 0.12 a	0.45 ± 0.07 a	24.03 ± 1.24 a	1.73 ± 0.87 a	2.99 ± 1.5 a	12.2 ± 3.75 a	5.2 ± 6.41 a	99.72 ± 50.76 a
D2	9.26 ± 0.09 a	2.78 ± 0.8 b	0.17 ± 0.08 a	0.37 ± 0.07 ab	24.12 ± 1.22 a	1.45 ± 0.64 ab	2.51 ± 1.11 ab	11.81 ± 3.55 a	2.28 ± 2.81 a	59.69 ± 19.03 ab
D3	9.29 ± 0.10 a	3.11 ± 0.7 b	0.16 ± 0.1 a	0.33 ± 0.06 b	24.38 ± 0.95 a	1.32 ± 0.96 b	2.27 ± 1.65 b	10.04 ± 5.67 a	1.39 ± 1.48 a	45.66 ± 18.76 b

Values are presented as mean ± SD (n = 8). Different letters within the same column indicate significant differences among soil depths at *p* < 0.05. pH (unitless); MC (%); TN, TP, TK, OC, OM (g·kg^−1^); AN, AP, AK (mg·kg^−1^).

**Table 2:** Correlations between soil physicochemical properties and microbial diversity indices and functional groups.

Trait	pH	MC	TN	TP	TK	OC	OM	AN	AP	AK
Shannon_b	−0.06	−0.25	−0.20	0.12	−0.21	−0.17	−0.17	−0.11	0.30	0.01
Simpson_b	−0.20	0.12	−0.04	0.42 *	−0.31	0.07	0.07	−0.03	0.34	0.16
Chao1_b	−0.11	−0.09	−0.09	0.23	−0.34	−0.01	−0.01	−0.03	0.34	0.08
Pielou_b	−0.27	0.16	0.15	0.45 *	−0.30	0.24	0.24	0.18	0.39	0.29
Shannon_f	−0.03	0.33	0.22	0.36	0.01	0.31	0.31	0.15	0.42	0.07
Simpson_f	−0.20	0.35 *	0.40 *	0.06	0.20	0.34	0.34	0.27	0.02	0.13
Chao1_f	−0.18	0.36	0.38	0.08	0.18	0.34	0.34	0.27	0.02 *	0.09
Pielou_f	−0.29	0.42	0.41	0.14	0.22	0.36	0.36	0.27	0.08	0.15
CH	−0.13	−0.25	−0.17	−0.12	−0.37	−0.04	−0.04	−0.12	0.22	−0.17
ACH	−0.07	−0.30	−0.24	−0.10	−0.43	−0.11	−0.11	−0.17	0.20	−0.20
US	0.05	−0.11	−0.06	0.42	−0.13	0.00	0.00	−0.06	0.08	−0.16
PPSW	−0.02	−0.29	−0.03	−0.25	−0.25	0.10	0.10	−0.06	0.36	−0.17

Asterisks (*) indicate statistically significant correlations at *p* < 0.05.

## Data Availability

The datasets presented in this article are not readily available because the data are part of an ongoing study. For access to the dataset, please contact the corresponding author.

## Abbreviations

The following abbreviations are used in this manuscript:

D1	0–20 cm soil depth
D2	20–40 cm soil depth
D3	40–60 cm soil depth
RDA	Redundancy Analysis
CCA	Canonical Correspondence Analysis
DCA	Detrended Correspondence Analysis
BRT	Boosted Regression Tree
TN	Total Nitrogen
AN	Alkali-Hydrolyzable Nitrogen
TP	Total Phosphorus
AP	Available Phosphorus
TK	Total Potassium
AK	Available Potassium
OC	Organic Carbon
OM	Organic Matter
MC	Moisture Content
ASV	Amplicon Sequence Variant
PERMANOVA	Permutational Multivariate Analysis of Variance
VIF	Variance Inflation Factor
PDP	Partial Dependence Plot
CH	Chemoheterotrophy
ACH	Aerobic Chemoheterotrophy
US	Undefined Saprotroph
PPSW	Plant Pathogen–Saprotroph–Wood Saprotroph

## Appendix A

**Table A1:** Methods for determining soil physicochemical properties.

Parameter	Analytical Method	Reference Standard
pH	Potentiometric method (1:2.5 soil-to-water suspension, glass electrode, deionized water).	HJ 962-2018 [58]
TN	Kjeldahl nitrogen determination (semi-micro distillation titration).	HJ 717-2014 [59]
AN	Alkali diffusion–colorimetric method.	LY/T 1228-2015 [60]
TP	Mo-Sb Anti spectrophotometric method	HJ 632-2011 [61]
AP	0.5 mol·L^−1^ NaHCO_3_ (pH 8.5) extraction–molybdenum–antimony colorimetric method (Olsen method).	NY/T 1121.7-2014 [62]
TK	HF–HClO_4_ digestion–flame photometry method.	NY/T 87-1988 [63]
AK	1 mol·L^−1^ NH_4_OAc (pH 7.0) extraction–flame photometry method.	NY/T 889-2004 [64]
OC	Determined as the difference between total carbon (TC) and inorganic carbon (IC) using an elemental analyzer.	——
OM	Calculated as OC × 1.724.	Soil Agricultural Chemical Analysis Methods [65]
MC	Oven-drying method at 105 °C to constant weight.	NY/T 52-1987 [66]
